# Supplementary material for: Investigating the Implementation of Community-Based Stroke Telerehabilitation in England; A Realist Synthesis Study Protocol
Source: Healthcare (Basel). 2024 May 15;12(10):1027. doi: 10.3390/healthcare12101027 (PMC11120767; doi:10.3390/healthcare12101027)
Supplement: Supplementary file 1 [file healthcare-12-01027-s001.zip › healthcare-2964886-supplementary file S1.pdf]

As part of the development of this protocol, a preliminary scoping of the evidence took place to inform our programme theory framework. The following databases were searched in October 2022: Medline (1996-2022), Embase (1980-2022), CINAHL (2000-2022), PsycInfo (2000-2022) and the Cochrane Library (December 2022). Searches were last updated on 02.12.22. The search had no language restrictions.

The full search strategy was devised in Medline (as shown below) and modified for other databases as appropriate. Search terms were developed to account for the variation in telerehabilitation definitions.

Examining telerehabilitation within the context of community-based stroke rehabilitation services yielded the following results: Medline (n=5), Embase (n=16), CINAHL (n=10), Psycinfo (n=1) (Cochrane=1). When combining stroke telerehabilitation terms with a realist methodological approach the following results were obtained: Medline (n=6), Embase (n=11), CINAHL (n=0), Psycinfo (n=1). A combination of terms related to realist reviews and stroke telerehabilitation in the community did not identify any studies. When search terms were introduced to explore implementation research literature in relation to stroke telerehabilitation, we found the following: Medline (n=1), Embase (n=5), CINAHL (n=5), Psycinfo (n=0).

Ovid MEDLINE(R) <1996 to November Week 4 2022>

Search terms Number of articles retrieved

1. stroke/ 124823
2. Stroke/ 154657
3. stroke.tw. 221893
4. (stroke or cerebrovasc* or brain vasc* or cerebral vasc* or cva* or apoplex*).tw. 261268
5. or/1-4 292031
6. rehabilitation.tw. 126404
7. 5 and 6 16349
8. exp stroke rehabilitation/ 16782
9. stroke rehabilitat*.tw. 3504
10. or/7-9 23392
11. ((early or earlier or post-discharge) adj5 (community or domiciliary or home or home-based) adj5 (rehabilitation or support* or care)).tw. 611
12. intermediate care.tw. 1276
13. 5 and 11 57
14. 5 and 12 50
15. (community stroke rehabilitation adj5 (based or services)).tw. 7
16. or/13-15 114
17. exp telerehabilitation/ 875
18. (virtual and care).tw. 6069
19. (telemedicine or telemetry or videoconferencing or telecommunications or remote consultation or
remote sensing technology).tw. 21546
20. (telemedicine or telemetry or tele-rehabilitation or telerehab or telehealth or tele-health or telehomecare or tele-homecare or telesupport or tele-support or telecoach or telecoaching or tele-coaching).tw. 25121
21. (telecommunication* or videoconference* or video-conferenc* or videoconsultation or video consultation or telestroke or teleconference* or tele-conference* or teleconsultation or tele-consultation
or telecare or ehealth or e-health).tw. 12302
22. (telespeech or tele-speech or teleOT or tele-OT or telepractice or teletherapy*).tw. 589
23. ((remote* or distance* or distant) adj5 (rehabilitation or therap* or treatment or physio* or occupational therap* or communication or consultation or education or support)).tw. 11007
24. (telespeech or tele-speech or teleOT or tele-OT or telepractice or teletherapy*).tw. 589
25. (virtual reality or virtual environment* or technolog*).tw. 442063
26. ((cell* or smart* or mobile or android or internet or web) adj3 (comput* or device or app* or phone)).tw. 124204
27. (smartphone or text-messag* or tablet) adj3 (device* or comput*)).tw. 2051
28. (mhealth or m-health or m health or mobile health).tw. 5723
29. activity tracker/ or exp accelerometry/ 12375

30. or/17-29 605683
31. 10 and 30 2089
32. 10 and 16 and 30 5
33. (Realist* and Synthes*).mp. 1646
34. (Realist* and Review*).mp. 7849
35. (realist or realistic evaluation).mp. 1915
36. or/33-35 9583
37. 30 and 36 1118
38. 10 and 30 and 36 6
39. 10 and 16 and 30 and 36 0
40. (qualitative adj2 (research or study)).tw. 65900
41. ((qualitative or interview) adj2 (research or study)).tw. 68883
42. ((qualitative or interview*) adj3 (research or stud*)).tw. 91713
43. or/40-42 91713
44. 10 and 30 and 43 46
45. Implementation.mp. 249723
46. (research adj3 (health services or implementation)).tw. 9011
47. (barriers adj3 (facilitators or enablers)).mp. 14166
48. or/45-47 262181
49. 10 and 30 and 48 147
50. 10 and 16 and 30 and 48 1

We also searched grey literature through:

1. The ProQuest database of dissertations and theses, which identified 19 results on stroke telerehabilitation but none involving realist methodology.
2. The NIHR Funding and awards database (1 completed study)
3. Stroke related charities i.e. The Stroke association, Different Strokes, British Heart Foundation.

Both the database and grey literature searchers confirmed that the proposed study has not been conducted before and is original in its aims and methodological approach.
